# Combined effect of compressing fresh concrete and curing regime on bonding performance of high strength repair mortar

**DOI:** 10.1016/j.heliyon.2024.e40242

**Published:** 2024-11-08

**Authors:** Parisa Bahri, Mahmoud Naderi

**Affiliations:** Department of Civil Engineering, Imam Khomeini International University, Qazvin, Iran

**Keywords:** High strength repair mortar, Twist-off method, Friction-transfer method, Wet sack curing, Steam curing, Compressing technique

## Abstract

High strength concrete (HSC) is highly appropriate for the retrofitting and rehabilitation of reinforced concrete structures due to its low permeability and high bonding strength. However, its low workability and sensitivity to curing conditions pose significant challenges for its implementation in such projects. This study introduces a novel technique to overcome the workability barrier of HSC while enhancing its bonding strength under various curing conditions. To achieve this, four different pressures (0, 0.87, 2.61, and 5.23 MPa) were applied to fresh high strength repair mortar (HSRM) and HSC for durations of 2, 4, and 8 h. Subsequently, different curing methods, including the wet sack method, steam curing method, and the use of curing compounds, were employed. The modulus of elasticity and compressive strength of HSC, in addition to bonding performance between substrate HSC and 28-day compressed HSRM, were evaluated using the “friction-transfer” and “twist-off” techniques, which are standard techniques for measuring the bonding strength of concrete both in the laboratory and in-situ. Also, to further assess the effect of curing regime and pressure on the microstructures of HSRM, SEM images of 28-day HSRM specimens were collected. The results indicated that increasing the pressure to 5.23 MPa and extending the duration to 8 h enhanced the ultimate torsional shear strength between HSRM and substrate HSC by an average of 174%. Additionally, the mechanical properties of HSC were considerably enhanced by applying 5.23 MPa pressure over 8 h. It is worth mentioning that the bonding strength between HSRM and substrate HSC without curing was highly affected by the duration of pressure application compared to the three investigated curing methods. The wet sack curing method was found to be the most effective for enhancing the degree of hydration and increasing the failure torsional shear stress of compressed HSRM specimens.

## 1 Introduction

Concrete structures can degrade due to several chemical and physical causes, leading to unserviceability of the structures [[1], [2], [3]]. For instance, the cover concrete of several bridge piers can be partially washed away and eroded by chloride ions, causing the first layer of rebar to fall off [4]. Additionally, the cover concrete and first layer of rebar can be damaged in concrete structures exposed to fire [5]. Therefore, in such cases, the damaged reinforced concrete structures must be repaired and bonded to prevent further degradation and ensure suitable serviceability [6]. The bonding strength and durability of substrate and repair concrete are crucial issues, and several studies have identified factors that enhance them [7,8]. One novel solution for enhancing the bonding strength between substrate and repair concrete involves using high-strength concrete (HSC) as repair concrete [[9], [10], [11]].

Despite the prompt developments in concrete industry, concrete with a higher compressive strength than 40–60 MPa is regarded as high strength concrete. According to the ACI committee [12], HSC are defined as concrete with 28-daycylinder compressive strength of more than 41 MPa. HSC offers numerous advantages over conventional concrete, and can be successfully implemented in compression members such as piles and columns. The use of high-strength materials in the construction industry enables the design of smaller components and reduces dead loads, leading to more efficient use of space in structures and, ultimately, more economical designs [[13], [14], [15]]. Additionally, the techniques essential for producing HSC create a compacted microstructure, making it difficult for aggressive materials to penetrate the concrete. This significantly enhances the long-term performance and durability of the structure. These benefits make HSC potentially appropriate for the retrofitting and rehabilitation of reinforced concrete (RC) structures [7]. Furthermore, the low permeability of HSC, in addition to its substantial mechanical properties, makes it ideal for strengthening and rehabilitating zones where the RC structure is subjected to harsh environments and heavy loads [16,17]. Other elements of the RC structure can be built with conventional concrete, as these elements are exposed to normal condition [18]. This attitude considerably enhances the structural performance, such as durability and costs of the rehabilitated RC structures [[19], [20], [21]].

The bond strength between old and new concrete commonly is a weak point in rehabilitation of RC structures [22,23]. The mechanical properties of the repair layer and its compatibility with substrate concrete play an important role in the performance of concrete structure [24]. Therefore, the ultimate shear strength of the repair bond should be enhanced to avoid debonding from the substrate concrete [25,26]. Júlio et al. [27] experimentally investigated the bond strength between repair and substrate concrete with various compressive strengths, and found that bond strength was enhanced as the compressive strength of repair concrete increased, with a new failure mode of monolithic [28]. Accordingly, HSC can be implemented as a repair concrete due to its strong bond with the substrate material. However, careful selection of ingredients, accurate mix proportioning, and proper curing are essential [[29], [30], [31]]. The ingredients and mix proportioning of HSC should be chosen to allow the repair layer to be easily poured and compacted without segregation [32]. The most critical factor in the mix design for achieving HSC is the water-to-cement ratio [33]. Conventional mixing methods typically use water-to-cement ratios between 0.22 and 0.40, leading to a 28-day compressive strength of approximately 60–130 MPa when utilizing normal density aggregates [34]. The water quality for HSC does not have stricter requirements than that for conventional concrete, with potable water generally specified [34].

Often, in order to provide excellent workability for HSC, additional water is required [35]. However, the water content's influence on HSC's mechanical properties is crucial and should be carefully controlled to achieve the desired workability [36,37]. Extra water could drastically decrease the durability and strength of HSC, despite providing a higher slump [35]. The additional water evaporates slowly during aging, resulting in an interconnected porous structure that reduces mechanical properties [35].

On the other hand, reducing the water content in the cement paste results in more durable and stronger concrete; however, this also increases friction between the aggregate and cement particles [38]. This increased friction can make the fresh concrete mix stiffer, complicating handling and compaction, and heightening the risk of cold joints and weak areas in the structure [39]. A common solution to improve the workability of HSC is the addition of mineral additives and chemical admixtures, which significantly enhance the properties of hardened concrete, including higher compressive strength and modulus of elasticity, reduced water absorption, increased density, and improved ultrasonic pulse velocity [[40], [41], [42], [43], [44]]. Nonetheless, the relatively high cost of these materials and the need for stringent quality control complicate the design of concrete mixtures aimed at achieving specific workability levels. Therefore, developing a viable method that minimizes processing costs while ensuring high workability without compromising the mechanical and physical properties of HSC is vital.

Curing conditions for HSC are also vital, significantly influencing durability and early strength gain [31,45]. Proper curing methods are even more essential than the increasing use of supplementary cementing materials, as inadequate curing can raise the likelihood of drying and plastic shrinkage in the concrete [46]. Curing techniques can include steam curing, water curing, and the application of curing compounds, all of which aid in completing hydration reactions in the mix [30,[47], [48], [49]]. The internal humidity of the concrete, which directly affects its strength, is influenced by the adopted curing conditions [50]. The curing regime is closely related to the water-to-binder ratio. Due to the low water-to-cement ratio of HSC, it is prone to self-desiccation, leading to increased shrinkage and micro-crack formation [51].

Methods like water and air curing can enhance the porosity of HSC, while steam and autoclave curing are favorable laboratory techniques that typically result in lower porosity [52]. Studies have shown that HSC cured in an autoclave for 360 min achieves compressive strength comparable to HSC air-cured for 28 days [53]. Steam curing accelerates cement hydration, enhancing early strength and promoting the production of hydration products [[54], [55], [56]], but it can negatively impact late strength and durability due to thermal damage [[57], [58], [59]]. Higher steam curing temperatures can hasten the hydration reaction, making early hydration heat more critical, which increases the overall degree of hydration [60,61]. However, this rapid formation of calcium hydroxide and hydrated calcium silicate can lead to uneven pore distribution, resulting in increased interconnectivity of pores and weakened structural integrity of the hydration products [62,63]. This may reduce the impermeability and compressive strength of the concrete [64].

While steam curing offers initial strength gains, it can adversely affect later strength due to an increase in pore size [30,65,66]. Research confirms that high-temperature steam curing aids initial strength development, but prolonged high curing temperatures can detrimentally affect the long-term strength of HSC [67]. Conversely, lower curing temperatures can lead to slower compressive strength growth rates [68]. Ambient and air curing can occur under normal weather conditions without the need for covering or soaking. Bushlaibi [69] and Bushlaibi & Alshamsi [70] indicated that dry open curing conditions may limit strength development in HSC, advocating for wet sack curing—where regular watering is applied—as the optimal method for strength attainment, particularly in arid climates.

This method helps reduce evaporation rates from the covering and is often utilized in resource-limited projects or remote areas [71]. The duration of wet curing, generally ranging from 2 to 7 days, may not significantly impact the durability of HSC in small sample evaluations [72]. Recommendations favor wet curing over dry methods for maximizing strength gains [73]. Wet sack curing is also beneficial for reducing autogenous shrinkage in high-performance concrete [74]. Table 1 summarizes different curing conditions for HSC. Various curing methods such as steam, hydrothermal, autoclave, microwave, and isothermal curing have been extensively studied, with steam curing being the most researched due to its positive effects on mechanical and durability properties. However, wet sack curing, curing compounds, and ambient curing have received less attention in the literature.

**Table 1:** Summary of different curing regimes of HSC.

Ref.	Year	Additives	Curing regime	W/b	Description
[30]	2021	Ultrafine palm oil fuel ash	Steam curing at 50, 65 and 80 °C	0.27	Strength improves within the first three days. Strength gains are lower at later ages. Best results were obtained at 80 °C for 16 h.
[47]	2023	Fly ash, Silica fume, Blast furnace slag	Hydrothermal and steam curing at 35 and60 °C	0.22	Strength increases at an early age. The rate of strength development decreases over time.
			Autoclave curing at 190 °C and 1.2 MPa,		Autoclave curing resulted in lower strength than hydrothermal.
[75]	2014	Blast furnace slag	Steam curing at 50 °C	0.33	Reduction in shrinkage and enhancement of early strength.
[45]	2019	Fly ash, Silica fume	Steam curing at 30 and 50 °C, Oven curing at 30 and 50 °C.	0.3, 0.35, 0.4	W/B Ratio Impact: Better strength noted for mixes with a 0.35 water-to-binder ratio. Steam Curing Comparison: 50 °C steam curing produced better overall compressive strength than 30 °C. Hardness in Steam Cured Specimens: Lower hardness observed in specimens with w/b ratios of 0.3 and 0.35. Improved Hardness: Higher hardness for 0.4 w/b ratio at 50 °C steam curing. Chloride Resistance: Lowest permeability at 50 °C steam curing with a 0.35 w/b ratio. Flexural Strength: Only increases with 50 °C oven curing at 0.35 and 0.40 w/b ratios. Chloride Resistance in Oven Curing: Decreases with 50 °C oven curing at these w/b ratios.
[76]	2006	Fly ash, Silica fume, Slag	Steam curing at 90 °C, Autoclave curing at 210 °C and 2 MPa	0.15	Steam Curing Duration: Best compressive strength improvement with 12-day steam curing. Strength Comparison: 12-day steam curing results in lower strength than autoclave. Autoclave Effectiveness: Best compressive strength improvement achieved in 8 h of autoclave curing.
[77]	2014	–	Microwave curing with 170–850 Watt power output	0.41	Highest water loss and strength at 850 W for 30 min.
[78]	2017	Fly ash, Silica fume, Slag	Steam curing at 80 °C), Autoclave curing at 175 °C and 0.9 MPa	0.27	28-Day Strength: Lower strength observed from steam curing after 28 days. Role of SCMs: SCMs help counteract the reduced strength from steam curing. Chloride Penetration: Increases, but silica fume can reduce it. Autoclave and SCMs: Slightly better compressive strength with fly ash as SCM in autoclave curing. Chloride Ingress: Higher increase observed in autoclave curing compared to steam.
[31]	2018	Fly ash, Silica fume, Slag	Steam curing at 50 °C, Steam curing in adiabatic condition	0.3	Early Strength Gain: Improvements noted in early strength. Chloride Permeability: Reduced only when SCMs are included.
[79]	2011	Fly ash, Slag powder, Nano CaCO_3_	Low temperature curing at 6.5 ± 1 °C	0.29	General reduction in compressive strength observed.
[80]	2014	Fly ash, Blast furnace slag	Isothermal curing at 10–30 °C	0.20, 0.30, 0.40	Effect of Temperature: Higher compressive strength at 30 °C for FA and BFS mixes; reduction in only cement-based mixes. Shrinkage and W/B Ratio: Higher shrinkage with lower water-to-binder ratios.
[81]	2016	–	Isothermal at 20 °C, Isothermal at 45 °C in adiabatic condition	0.33	Curing Temperature: Lower shrinkage for specimens cured at 20 °C. Cracking Stress: Higher cracking stress to tensile strength ratio at 20 °C curing. Cracking Resistance: Better resistance at 20 °C than 45 °C and adiabatic curing. Cooling Effect: Cracking occurs earlier in cooling under restrained conditions.
[65]	2016	Fly ash, Recycled aggregate	Steam curing at 65 °C	0.28	Recycled Aggregates: Reduced pore size and porosity observed after steam curing. Strength Reduction: Lower reduction in strength and modulus for recycled aggregates with steam curing.
[82]	2022	Silica fume, Quartz flour	Autoclave curing at 220 °C and 2.1 atm	0.2, 0.25, 0.30, 0.35, 0.40	Benefits of Low W/B Ratios: Improved strength for water-to-binder ratios below 0.30 due to enhanced pozzolanic reactions. Porosity Increase: Noted in autoclave cured mixes with 0.35 and 0.40 w/b ratios.

## 2 Research gap and novelty of the study

The literature reveals a lack of suitable methods for enhancing the workability and bonding strength of HSC under various curing regimes. This study presents a novel method that overcomes existing challenges without the need for additives, external vibrations, or workability control. The approach involves compressing freshly cast High-Strength Reactive Mixture (HSRM) using a specially crafted pressure device. This technique prevents segregation, bleeding, and workability loss during casting. As the mixture is compressed, excess water is gradually removed, significantly lowering the water-cement ratio and porosity.

For this purpose, different pressures (0 MPa, 0.87 MPa, 2.61 MPa, and 5.23 MPa) were applied to fresh HSRM for varying durations (2, 4, and 8 h). Then, the specimens were exposed to various curing regimes. Based on the literature, steam curing is the most investigated method for HSC, yielding acceptable results for its mechanical and durability properties. Conversely, wet sack curing, the use of curing compounds, and ambient curing have been rarely explored. Therefore, this study investigated steam curing, wet sack curing, ambient curing, and the use of curing compounds to identify the best method for maximizing the bonding strength between substrate HSC and compressed HSRM.

Finally, the combined effect of compressing fresh HSRM and varying curing conditions on the bonding strength between HSC and HSRM was evaluated using twist-off and friction-transfer methods. The twist-off method is simple, cost-effective, and accurate, with wide applicability on both horizontal and vertical surfaces for evaluating concrete bond performance without the need to train workers [83]. Additionally, the friction transfer method is a rapid and straightforward technique for controlling in-situ concrete quality, evaluating de-molding time, and measuring the quality of repair methods in different weather conditions [84]. Also, to additional assessment of the effect of curing regime and pressure on the microstructures of HSRM, SEM images of 28-day HSRM specimens were collected.

## 3 Experimental program

### 3.1 Materials

For all specimens, Type II Portland cement was used in both HSRM and substrate HSC [85]. Chemical compositions and physical properties of the cement are presented in Table 2. Additionally, potable water free of CO_2_ and impurities was utilized for HSRM and substrate HSC. According to ASTM C 494, a poly-carboxylate ether-based superplasticizer (specific weight = 1.23 kg/l) was used to reach the favorite workability in specimens with a W/C ratio of 0.35 [86]. Micro silica with purity of 99.9% was also incorporated, with its chemical compositions listed in Table 2.

**Table 2:** Chemical compositions and physical properties of type II Portland cement and micro silica.

Chemical components	Cement	Micro silica
SiO_2_ (%)	20.90	95
Al_2_O_3_ (%)	5.35	1.32
Fe_2_O_3_ (%)	4.71	0.87
CaO (%)	63.03	0.49
MgO (%)	1.58	0.97
SO_3_ (%)	2.14	0.1
Na_2_O (%)	0.25	0.31
K_2_O (%)	0.66	1.01
CL (%)	–	0.04
P_2_O_2_ (%)	–	0.16
H_2_O (%)	–	0.08
C (%)	–	0.3
SiC (%)	–	0.5
LOI (%)	1.38	–
C_3_S	69.27	–
C_2_S	9.81	–
C_3_A	6.68	–
C_4_AF	14.24	–
Physical properties		
Density (gr/cm^3^)	3.286	2.255
Specific surface (m^2^/kg)	3275	>20000
Autoclave expansion (%)	0.21	–
Initial setting time (min)	110	–
Final setting time (min)	190	–

**Fig. 1:**
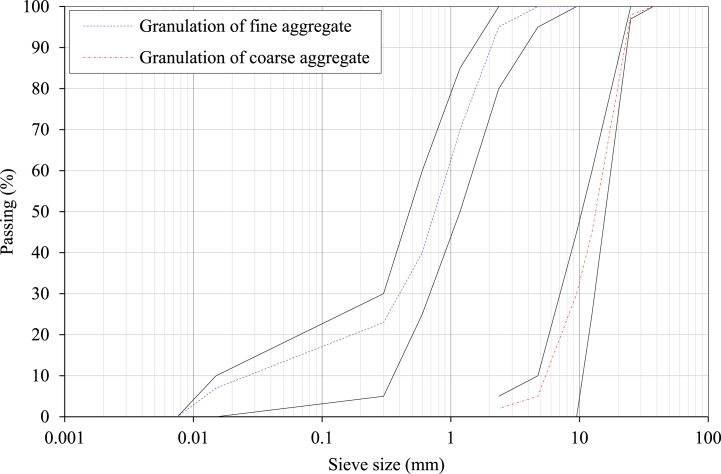
The grading curve of aggregates.

For the substrate HSC, crushed stone and rounded washed sand were employed as the coarse and fine aggregates, respectively, while rounded washed sand was used as the fine aggregate in HSRM. The specific gravity of fine and coarse aggregates in their saturated surface dry (SSD) state was 2.52 and 2.55, respectively, and their water absorption rates were 1.65% and 2.50%, respectively [87,88]. Based on ASTM C136-01, the grading curve of aggregates is shown in Fig. 1 [89].

### 3.2 Mixture design and preparation

The water-to-cement ratio was maintained at 0.35 for both HSRM and substrate HSC. All replacements were performed by mass. The slump values of substrate HSC and HSRM were maintained at 9 ± 1 cm and 14 ± 1 cm, respectively, according to the ASTM C 143/C 143M–03 standard [90]. Table 3 shows the mix designs of HSRM and substrate HSC.

**Table 3:** The mixt designs of HSRM and substrate HSC.

Mixture code	W/C	Cement (kg/m^3^)	Water (L/m^3^)	Fine Aggregate (kg/m^3^)	Coarse Aggregate (kg/m^3^)	Micro silica (kg/m^3^)	Superplasticizer (kg/m^3^)
Substrate HSC	0.35	530	185.5	700	1030	37	4.7
HSRM	0.35	700	245	1530	0	37	4.7

For specimen preparation, the substrate HSC specimens were prepared first. Dry components (fine and coarse aggregates and cement) were mixed for 1 min, following which water, micro silica, and superplasticizer were gradually added and mixed for 4 min. Proper mixing was followed by a slump test on the fresh concrete as per the standard [90]. Fresh concrete was then cast into 15 cm × 15 cm × 15 cm molds. After vibration, the substrate HSC specimens were cured with a wet towel at laboratory temperature for 24 h. Subsequently, they were demolded and submerged in a saturated limewater at 21 ± 3 °C for 28 days. Finally, the substrate HSC specimens were cut into 15 cm × 15 cm × 5 cm slabs, and their cutting surfaces were smoothed to minimize surface friction.

**Fig. 2:**
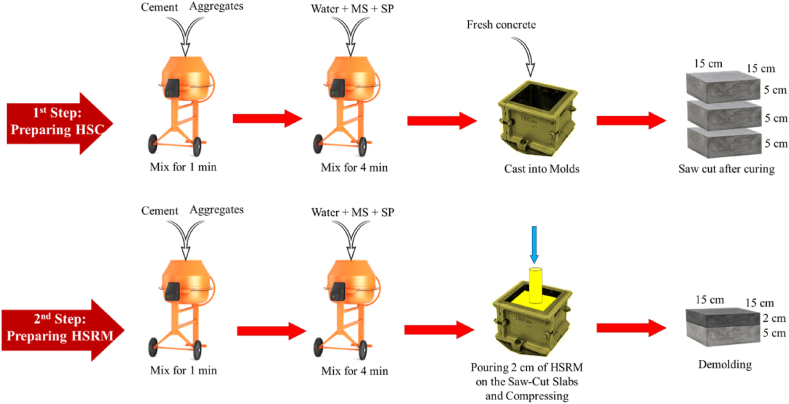
Schematic process of preparing substrate HSC and HSRM.

**Fig. 3:**
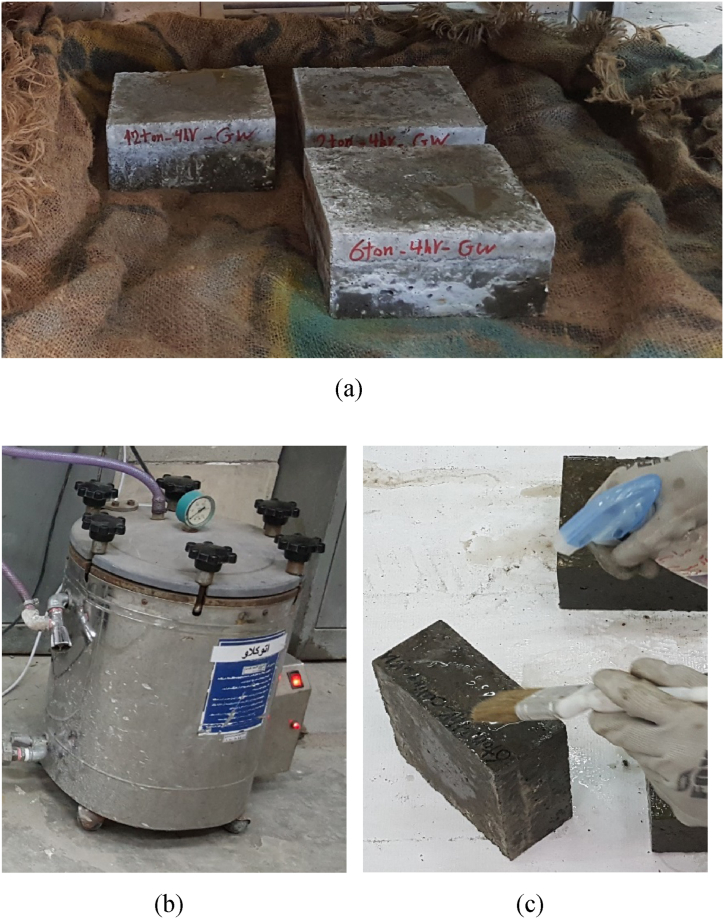
Curing substrate HSC and compressed HSRM using (a) wet sack, (b) steam, (c) curing compound.

Next, a 2 cm-thick layer of HSRM was applied to the saw-cut surfaces of these slabs. Cement and fine aggregates were mixed for 1 min, followed by the gradual addition of superplasticizer, water, and micro silica, which were then mixed for 4 min. After proper mixing, a slump test was conducted on the fresh HSRM [90]. The fresh HSRM was then poured onto the cast slabs and subjected to four different pressures for 2, 4, and 8 h. The schematic process of preparing substrate HSC and HSRM is illustrated in Fig. 2. After unloading, the fresh HSRM specimens were cured with a wet sack at laboratory temperature for 24 h. Subsequently, the substrate HSC and compressed HSRM were cured under three different conditions: wet sack method (Fig. 3(a)), steam curing method for 20 h (Fig. 3(b)), and curing compound application (Fig. 3(c)). A polyolefin emulsion-based curing compound with a density of 0.9 ± 0.02 g/cm³ was used according to ASTM C309 [91].

To assess the combined effect of pressure and curing regimes, the modulus of elasticity and compressive strength of HSC under different pressures and curing regimes were initially assessed. The procedure for preparing these specimens for initial evaluation was akin to that of repair mortar. After mixing the HSC ingredients, the fresh HSC was poured into 15 cm × 15 cm × 15 cm molds and subjected to four different pressures for 2, 4, and 8 h. After unloading, the HSC specimens were cured under the three aforementioned conditions.

### 3.3 Instrumentation

**Fig. 4:**
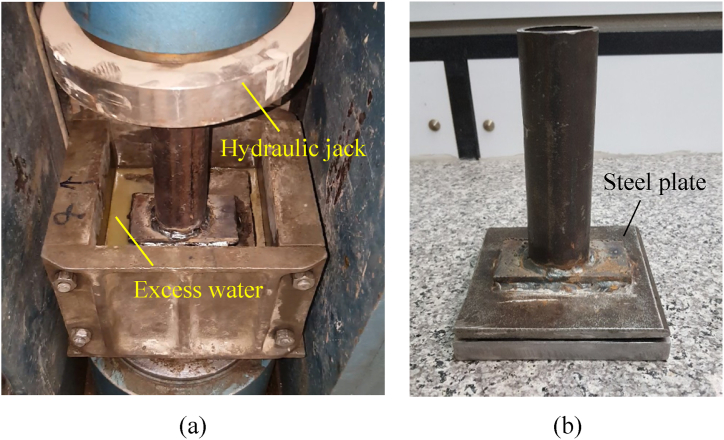
(a) Applying pressure on HSRM and expelling excess pure water, (b) Apparatus for transferring pressure uniformly.

To assess the effect of compressive stress on the bonding strength between HSRM and substrate HSC, specimens were subjected to four different pressures (0, 0.87, 2.61, and 5.23 MPa) for durations of 2, 4, and 8 h. The pressure apparatus used in this study was a hydraulic jack that applies a specified force to the fresh HSRM for a designated period, as shown in Fig. 4(a). The specimens were placed at the midpoint of a steel plate, and compression force was gradually and uniformly transferred to the specimen surface using the hydraulic jack (Fig. 4(b)). To separate the steel plate from the fresh HSRM, a 15 cm × 15 cm binding cover was laid on top of the HSRM. As the pressure was applied, a small amount of water, added solely for achieving the desired workability, was gradually expelled from the edges of the steel plate. As shown in Fig. 4(a), the expelled water from the compressed HSRM specimens was very pure and free from other materials.

### 3.4 Testing procedure

To assess the combined effect of pressure and curing conditions, modulus of elasticity and compressive strength of HSC specimens under various pressures and curing conditions were initially evaluated. The modulus of elasticity and compressive strength tests were performed on 15 cm × 15 cm × 15 cm HSC specimens, with three specimens made for each condition to obtain average values. After evaluating these combined effects, repair specimens were prepared for semi-destructive tests.

**Fig. 5:**
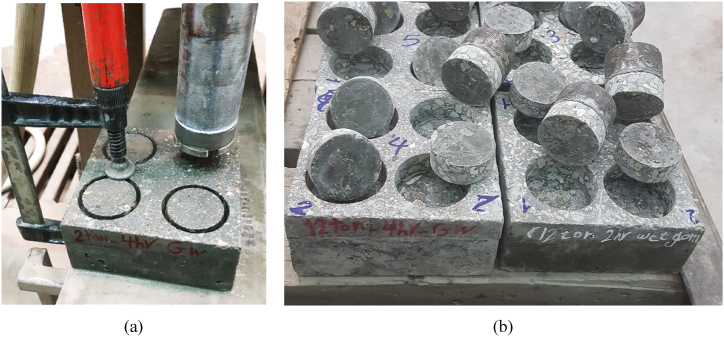
(a) Core drilling the compressed HSRM, (b) Fracture of partial cores within the bond.

Semi-destructive methods are commonly employed to evaluate various properties of concrete components. These methods inflict minor damage over a limited area, ensuring that repairs are superficial and do not significantly compromise the structural integrity. In this study, the bond performance between compressed HSRM and substrate HSC was evaluated using “twist-off” and “friction-transfer” methods [83,84]. Prior to testing, specimens were dried under laboratory conditions for 48 h. Partial cores, 25 mm deep and 50 mm in diameter, were drilled into the compressed HSRM of each slab using ordinary diamond-tipped drills (Fig. 5(a)). The specimens were then securely fixed to a working table, where the twist-off and friction-transfer tests were conducted. The depth of the partial core was sufficient to penetrate into the substrate HSC, and it is important to note that fractures occurred along the bond between the substrate HSC and compressed HSRM. In both tests, the fracture of the partial core exhibited a zero-degree angle relative to the horizontal plane (Fig. 5(b)).

In the twist-off test, the surface of the specimens was thoroughly abraded with coarse sandpaper to enhance the bond strength by increasing the cross-sectional area of the epoxy resin with respect to the specimens [83]. Then, a circular metallic probe was adhesively bonded on the partially drilled core, allowing the epoxy resin to cure completely for 24 h. The epoxy resin was selected based on ASTM C881/C881M − 02 [92]. The physical and chemical properties of the epoxy resin used are provided in Table 4.

**Table 4:** The chemical and physical properties of the employed epoxy resin.

Solids (Wt.%)	100 %
7-day Compressive strength (MPa)	70 MPa
Pot life	90 min at 25 °C 4 h at 35 °C
Drying time (hour)	10 h at 25 °C 4 h at 35 °C
Over coating time (hour)	After 24 h curing
Color	Grey
Chemical resistance	Resistant to fats, oil, petrol, salt and sugar solution, dilute mineral acids, bleach, alkalis, white spirit. Not resistant to xylene.

**Fig. 6:**
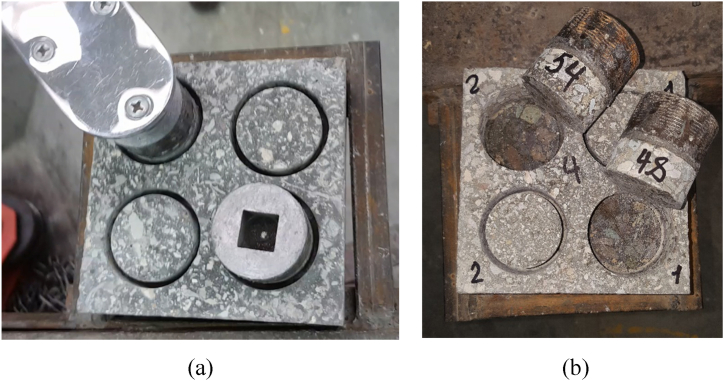
(a) Installing torque-meter on the metal probe (b) Fracture of partial cores within the bond in the twist-off test.

Torque was manually applied and gradually increased using a standard torque meter attached to the metal probe (Fig. 6(a)). Due to the significantly higher strength of the epoxy adhesive layer compared to the torsional resistance of the bond between the substrate concrete and compressed HSRM, fractures occurred within the bond (Fig. 6(b)). The ultimate torque was measured, and the failure torsional shear stress (MPa) was obtained using Eq. (1).

(1) $$\tau_{\left(\text{failure}\right)}=\frac{T_{\left(\text{failure}\right)}r}{J}=\frac{2T_{\left(\text{failure}\right)}}{\pi r^{3}}$$

Where $T_{\left(\text{failure}\right)}$ is the ultimate torque (N.mm); *r* is the radius of partially drilled core (25 mm); and *J* is the second polar moment of area.

**Fig. 7:**
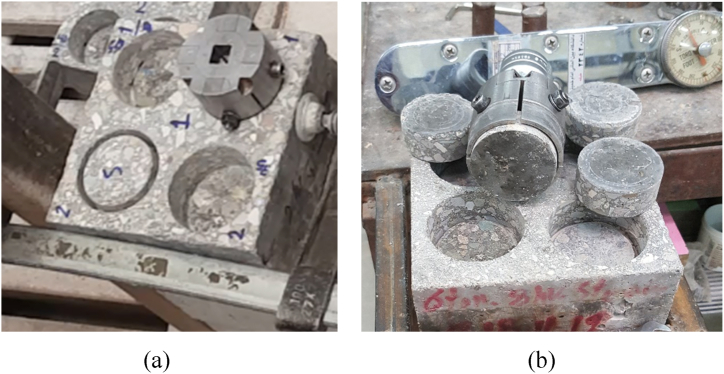
(a) Installing metallic gripping device on the partially drilled core (b) Fracture of partial cores within the bond in the friction-transfer test.

For the friction-transfer test, a metallic gripping device was secured onto the partially drilled core. A manually applied and gradually increasing torque was applied with a torque meter positioned on the gripping device (Fig. 7(a)) [84]. Similar to the twist-off test, the gripping device's frictional resistance was stronger than the torsional resistance of the bond between the substrate HSC and the compressed HSRM, leading to fractures within the bond. (Fig. 7(b)). The failure torsional shear stress (MPa) was also determined using Eq. (1).

## 4 Results and discussion

This study aims to assess the combined impact of pressure and curing conditions on the bonding performance between substrate HSC and compressed HSRM by employing semi-destructive methods such as “twist-off” and “friction-transfer” tests. Additionally, it evaluates the modulus of elasticity and compressive strength of HSC under various pressures and curing conditions. A summary of the results for the modulus of elasticity and compressive strength tests on HSC specimens is presented in Table 5, while Table 6 summarizes the twist-off and friction-transfer tests conducted on HSRM specimens under different pressures and curing regimes. It is essential to highlight that the values in these tables represent the averages of three specimens.

**Table 5:** Compressive strength and modulus of elasticity of HSC under various pressures and curing conditions.

Test	Time of pressure (hr)	Pressure (MPa)	Results			
			Without curing	Wet sack curing	Curing compound	Steam curing
Compressive strength (MPa)	0	0	47.3	75.6	56.8	65.5
	2	0.87	53.6	87.7	64.4	74.3
		2.61	61.5	92.7	75.0	83.7
		5.23	62.1	95.6	75.6	87.3
	4	0.87	66.2	92.1	76.0	81.8
		2.61	71.7	97.8	85.2	91.2
		5.23	76.5	102.1	88.4	96.1
	8	0.87	72.1	93.2	88.4	89.4
		2.61	80.8	99.3	91.6	95.5
		5.23	85.6	105.1	94.4	100.3
E (GPa)	0	0	13.13	18.56	15.66	18.12
	2	0.87	14.05	19.86	16.76	19.39
		2.61	15.88	22.27	18.48	22.07
		5.23	16.56	23.41	19.46	22.67
	4	0.87	17.04	28.37	17.68	25.35
		2.61	19.00	30.92	20.15	27.44
		5.23	20.02	32.91	21.02	29.23
	8	0.87	18.19	28.37	18.31	25.95
		2.61	19.21	33.19	20.27	28.93
		5.23	20.23	34.19	21.77	30.42

**Table 6:** Bonding strength of HSRM under various pressures and curing conditions.

Test	Time of pressure (hr)	Pressure (MPa)	Results			
			Without curing	Wet sack curing	Curing compound	Steam curing
τ_(failure)_ in twist-off (MPa)	0	0	0.21	2.81	1.02	2.01
	2	0.87	0.68	2.97	1.61	2.28
		2.61	0.78	3.28	1.80	2.57
		5.23	0.8	3.45	1.89	2.68
	4	0.87	0.89	3.12	1.90	2.51
		2.61	0.96	3.56	2.08	2.80
		5.23	1.02	3.71	2.21	2.95
	8	0.87	0.94	3.23	1.96	2.59
		2.61	1.05	3.58	2.29	2.93
		5.23	1.11	3.85	2.36	3.08
τ_(failure)_ in friction transfer (MPa)	0	0	0.16	2.86	1.1	1.94
	2	0.87	0.65	2.91	1.40	2.07
		2.61	0.74	3.21	1.57	2.34
		5.23	0.76	3.38	1.65	2.44
	4	0.87	0.85	3.07	2.00	2.51
		2.61	0.92	3.50	2.18	2.80
		5.23	0.98	3.65	2.32	2.95
	8	0.87	0.87	3.18	2.00	2.68
		2.61	0.97	3.52	2.34	2.83
		5.23	1.02	3.78	2.41	2.98

### 4.1 Compressive strength and modulus of elasticity of HSC

As shown in Table 5, increasing both the pressure and the duration of application on HSC specimens led to improvements in modulus of elasticity and compressive strength. The peak values for these properties were observed in HSC specimens exposed to a pressure of 5.23 MPa for 8 h. However, it's crucial to note that both compressive strength and modulus of elasticity displayed significant increases up to 4 h of pressure application, after which the rate of increase gradually decreased over the following 4 h. Typically, the compressive strength of concrete is directly associated with its modulus of elasticity [93]. This trend can be explained by the enhanced packing density and improved interlocking of aggregate particles that result from reduced porosity under higher pressure and longer duration.

**Fig. 8:**
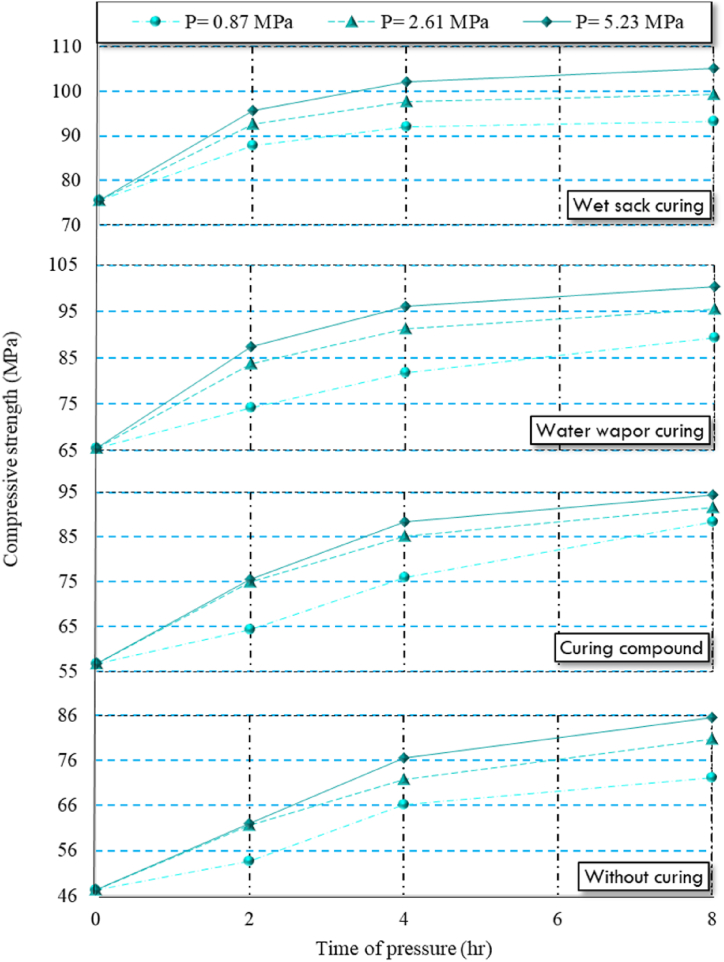
Compressive strength of compressed HSC specimens under different curing regimes.

**Fig. 9:**
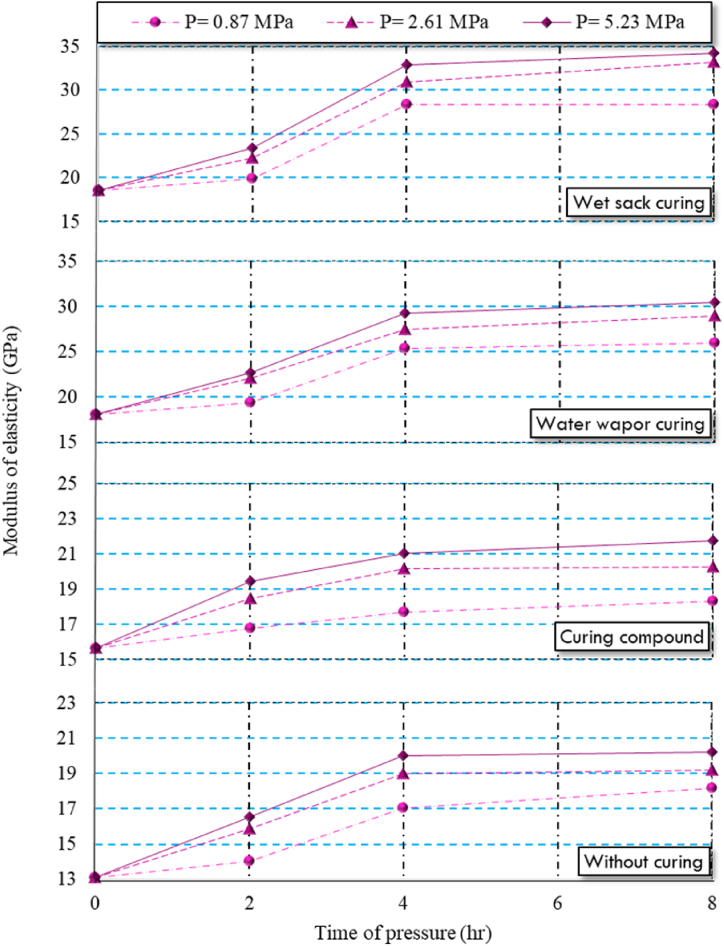
Modulus of elasticity of compressed HSC specimens under various curing regimes.

In compressed concretes, applied pressure forces out the excess water, which is free from cement particles. Maintaining constant pressure ensures complete removal of this water, enhancing both compressive strength and modulus of elasticity [94]. Essentially, reducing the water-to-cement ratio and lowering porosity increases the volume of solid phases and strengthens matrix bonds, thus improving compressive strength and elasticity. After complete water discharge, the pressure helps the aggregates interlock better, further boosting these properties [94]. Among factors enhancing mechanical properties, lowering the water-to-cement ratio is key to increasing concrete strength [95,96]. Fig. 8, Fig. 9 provide a visual representation of the modulus of elasticity and compressive strength, respectively. Notably, the increase in modulus of elasticity was minimal when extending the pressure duration from 4 to 8 h. Similarly, Nematzadeh and Naghipour [94] found that applying pressure on fresh concrete enhanced its mechanical properties. Additionally, the results illustrated that wet sack curing method was the most effective method for enhancing hydration and increasing both modulus of elasticity and compressive strength in compressed HSC specimens. Specifically, specimens subjected to wet sack curing exhibited compressive strength and modulus of elasticity values that were, on average, 1.4 and 1.6 times higher than those of specimens without curing, respectively. This method effectively maintained the water content in the HSC, promoting continued hydration reactions.

In comparison, steam curing and using curing compound yielded lower modulus of elasticity and compressive strength. Specimens under steam curing and those treated with a curing compound demonstrated compressive strength values that were, on average, 1.3 and 1.2 times higher than those of uncured specimens, respectively. As shown in Table 3, the water-to-cement ratio of HSC was 0.35, which hindered complete hydration when using the curing compound, leading to a significantly lower degree of hydration compared to the wet sack curing method.

The irregular structure of hydration products in steam-cured compressed HSC specimens contributed to slightly lower modulus of elasticity and compressive strength compared to specimens cured in wet sacks. Lastly, compressed HSC specimens without any curing exhibited the lowest compressive strength and modulus of elasticity.

### 4.2 Bonding strength of HSRM

As shown in Table 6, increasing the pressure and duration of pressure on HSRM specimens resulted in higher failure torsional shear stress, as observed in both twist-off and friction transfer tests. The maximum failure torsional shear stress occurred in HSRM specimens subjected to 5.23 MPa for 8 h. However, in both tests, the failure torsional shear stress increased significantly at a pressure duration of 2 h but then gradually decreased when the pressure was maintained for 8 h. This trend can be attributed to the reduced porosity and enhanced interlocking between aggregate particles resulting from the increased pressure and duration.

**Fig. 10:**
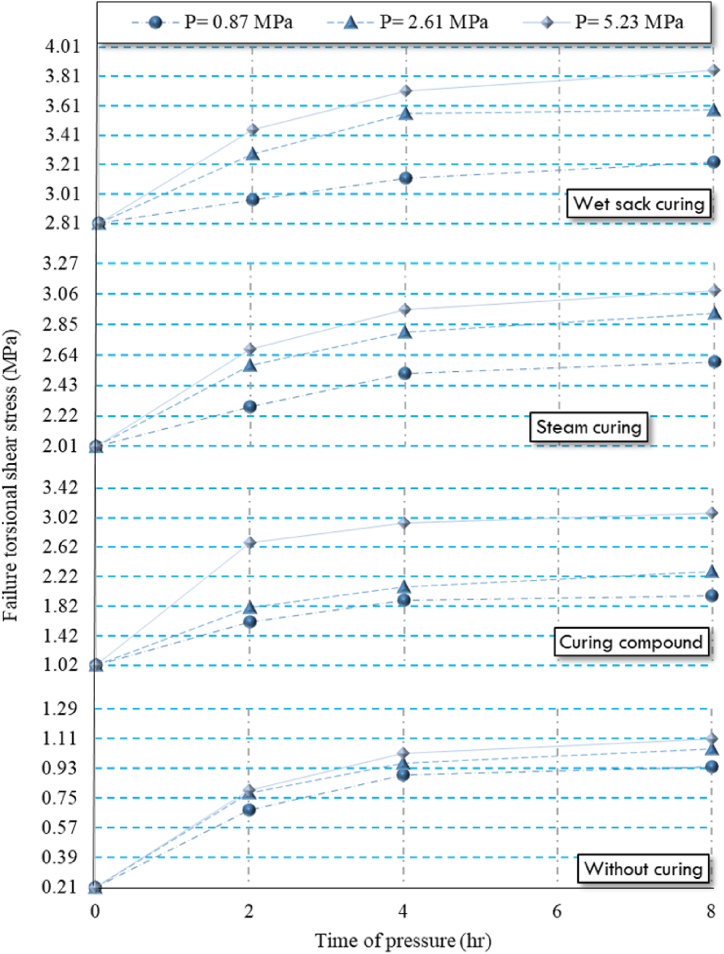
Twist-off results of compressed HSRM specimens under various curing regimes.

**Fig. 11:**
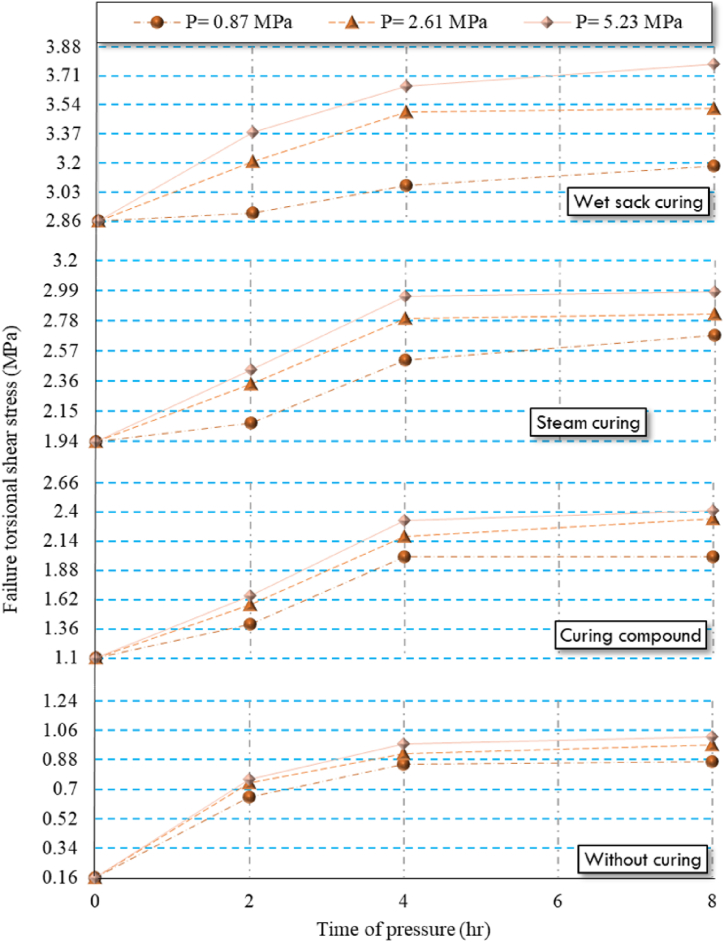
Friction transfer results of compressed HSRM specimens under various curing regimes.

As previously highlighted, the discharge of excess water reduces the water-to-cement ratio and concrete porosity, increases the solid phases volume, and improves matrix bonding, which culminates in higher bonding strength [94]. Once excess water is completely discharged, the pressure applied to the fresh concrete is absorbed by the aggregate, improving the interlocking between aggregate particles, thereby further enhancing bonding strength [94]. Research by, Nematzadeh and Naghipour [94] corroborates that applying pressure to fresh concrete leads to improved mechanical properties. Among the various factors influencing the mechanical properties of compressed concrete, the reduction of the water-to-cement ratio is particularly significant in augmenting the strength of various concrete types [95,96]. For a clearer understanding, the results of the failure torsional shear stress are illustrated in Fig. 10, Fig. 11.

It is noteworthy that the bonding strength between HSRM and substrate HSC was significantly affected by the duration of pressure, particularly concerning the use of curing compounds and in conditions without curing. Furthermore, for both steam curing and wet sack curing, the magnitude of the pressure also had a substantial effect on bonding strength.

Both twist-off and friction transfer tests indicated that wet sack curing was the most effective method for enhancing hydration levels and increasing the failure torsional shear stress of compressed HSRM specimens. Specifically, the results from both tests showed that specimens under wet sack curing had average values four times higher than those of uncured specimens. This method effectively maintains the water content in HSRM, facilitating continued hydration reactions and improving degree of hydration.

Conversely, steam curing and the use of curing compounds resulted in lower failure torsional shear stress for compressed HSRM specimens. Specifically, specimens under steam curing and curing compound had twist-off and friction transfer test results that were, on average, three and two times higher than those of uncured specimens, respectively. As mentioned in Table 3, the water-to-cement ratio of HRSM was only 0.35, which impeded the completion of hydration reactions when using a curing compound, leading to significantly lower degree of hydration compared to wet sack curing.

Finally, the irregular structure of hydration products in compressed HSRM specimens subjected to steam curing contributed to slightly lower failure torsional shear stress compared to those cured with wet sacks. Compressed HSRM specimens without any curing exhibited the lowest failure torsional shear stress.

## 5 Microstructural analysis

**Fig. 12:**
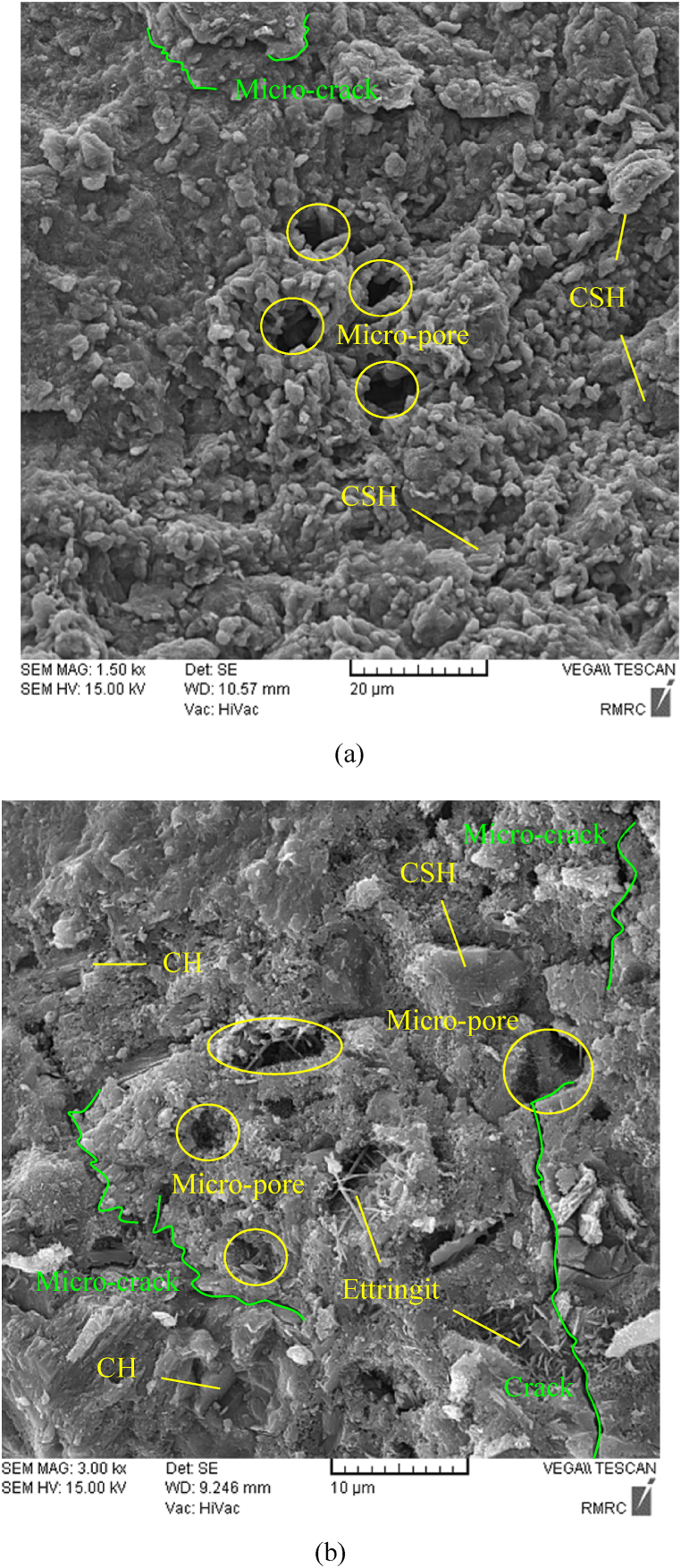
SEM images of uncompressed HSRM (a) under wet sack curing, (b) without curing.

To more analyze the effect of curing regime and pressure on the microstructures of HSRM, SEM images of 28-day specimens were collected. Fig. 12(a) and (b) depict the SEM images of uncompressed HSRM under wet sack curing and without curing, respectively. The presence of hexagonal platelets of CH can be perceived in the HSRM without curing. The reaction of CH and its transformation into CSH in 28-day HSRM are due to the development of hydration reactions. Given the presence of water in the wet sack curing method, which results in the development of hydration reactions during the early stages [[97], [98], [99]], it can be observed in Fig. 12 that the CH present in the HSRM without curing transforms to CSH in the HSRM under wet sack curing.

CSH, the major cement hydration product, is responsible for the strength of the cementitious matrix, consists of heterogeneous nanoporous materials with sheet structure containing calcium, coxygen atoms, and silicate tetrahedra. Unhydrated cement grains, together with CSH and other hydration products, form a complex porous structure that includes capillary and intra/inter CSH pores [100]. As shown, the length of micro-cracks in the HSRM without curing was on average shorter than in the HSRM under wet sack curing. Moreover, the microstructure of the HSRM specimen without curing was more porous than that under wet sack curing, with larger voids on average found in the specimen without curing. Due to the development of hydration reactions, the microstructure of the HSRM under wet sack curing was significantly denser than that of the HSRM specimen without curing.

**Fig. 13:**
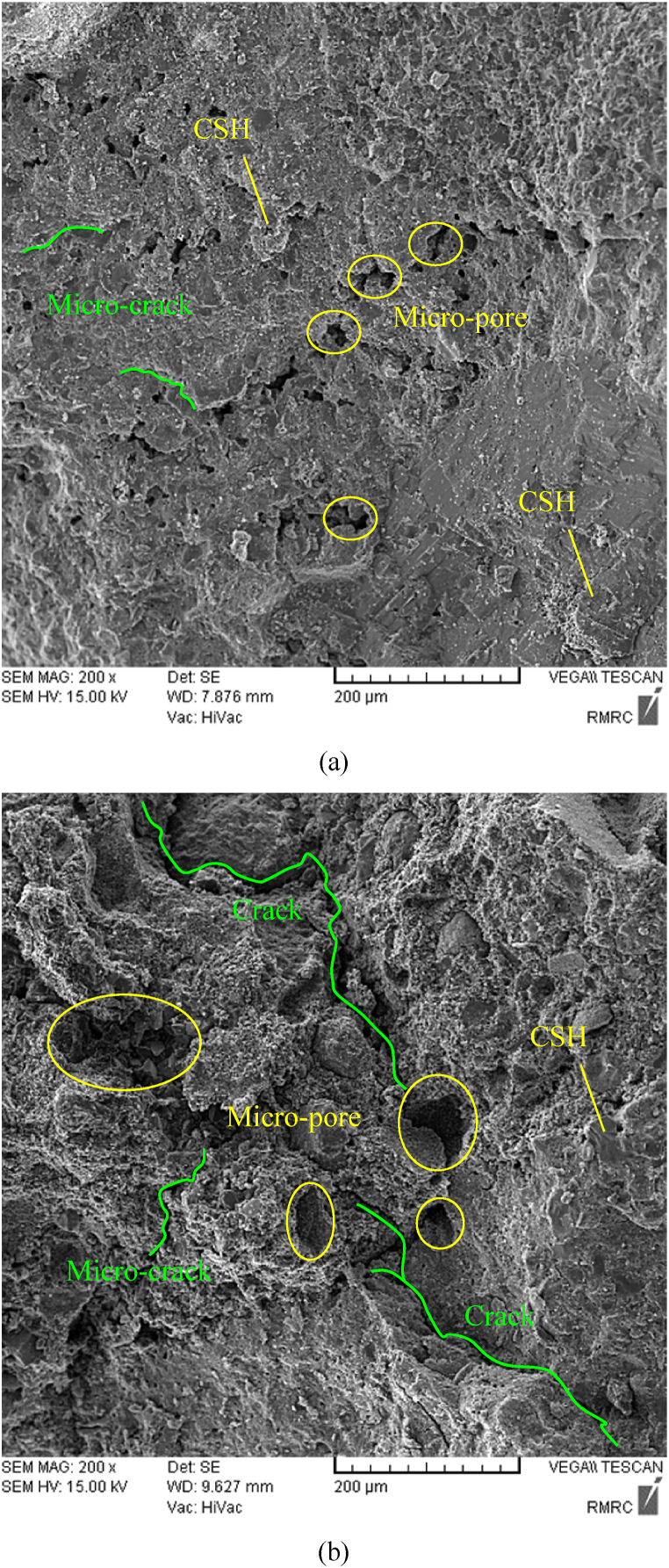
SEM images of HSRM under wet sack curing and pressure of (a) 5.23 MPa, (b) 0 MPa for 8 h.

Fig. 13(a) and (b) illustrate the SEM images of HSRM cured using a wet sack method, with one subjected to a pressure of 5.23 MPa and the other at 0 MPa for 8 h. In the case of compressed HSRM, the applied pressure helps gradually discharge excess water from the fresh concrete, enhancing its compactness. This increased pressure leads to a more compact microstructure in the concrete [94]. Furthermore, with the full discharge of excess water, the pressure on the fresh concrete is borne by the aggregates, resulting in improved interlocking between aggregate particles [94]. Under pressure, the water-to-cement ratio in HSRM is reduced [95,96], contributing to the differences observed in microstructure. Thus, the uncompressed HSRM specimen showcased a more porous structure than its compressed counterpart, with larger average voids. Moreover, the compressed HSRM exhibited shorter micro-cracks compared to the uncompressed version under wet sack curing. This indicates that the microstructure of the compressed HSRM under wet sack curing was notably denser than that of the uncompressed HSRM specimen.

## 6 Statistical analysis

The preparation of HSRM and the evaluation of bond performance between compressed HSRM and substrate HSC over different time periods are both time-consuming and costly operations. In this section, a statistical regression analysis was performed to predict the bond performance between compressed HSRM and substrate HSC, influenced primarily by curing conditions, pressure magnitude, and the duration of pressure application.

Initially, the relationship between these parameters and failure torsional shear stress was examined. As shown in Fig. 10, Fig. 11, a nonlinear relationship was observed between the parameters and the failure torsional shear stress for both compressed HSRM and substrate HSC. Therefore, using nonlinear regression analysis, regardless of the correlation between the parameters, Equations (2), (3)) were proposed to predict the failure torsional shear stress in both the twist-off and friction transfer tests.

(2) $$\text{Twist}-\text{off}:\tau_{\left(\text{failure}\right)}=\left\{ \begin{array}{cc} 0_{\cdot }205+0_{\cdot }098{\left(p\right)}^{0_{\cdot }508}+0_{\cdot }33{\left(t\right)}^{0_{\cdot }354}\;R^{2}=0_{\cdot }97 & \left(\text{Without}\;\text{curing}\right)\\ 2_{\cdot }761+0_{\cdot }211{\left(p\right)}^{0_{\cdot }742}+0_{\cdot }05{\left(t\right)}^{0_{\cdot }968}\;\left(R^{2}=0_{\cdot }93\right) & \left(\text{Wet}\;\text{sack}\;\text{curing}\right)\\ \begin{array}{c} 1_{\cdot }007+0_{\cdot }243{\left(p\right)}^{0_{\cdot }491}+0_{\cdot }30{\left(t\right)}^{0_{\cdot }489}\;\left(R^{2}=0_{\cdot }96\right)\\ 1_{\cdot }981+0_{\cdot }227{\left(p\right)}^{0_{\cdot }617}+0_{\cdot }10{\left(t\right)}^{0_{\cdot }762}\;\left(R^{2}=0_{\cdot }95\right) \end{array} & \begin{array}{c} \left(\text{Curing}\;\text{compound}\right)\\ \left(\text{Steam}\;\text{curing}\right) \end{array} \end{array}\right.$$

(3) $$\text{Friction}\;\text{transfer}:\tau_{\left(\text{failure}\right)}=\left\{ \begin{array}{cc} 0_{\cdot }156+0_{\cdot }098{\left(p\right)}^{0_{\cdot }484}+0_{\cdot }36{\left(t\right)}^{0_{\cdot }295}\;\left(R^{2}=0_{\cdot }96\right) & \left(\text{Without}\;\text{curing}\right)\\ 2_{\cdot }792+0_{\cdot }159{\left(p\right)}^{0_{\cdot }848}+0_{\cdot }03{\left(t\right)}^{1_{\cdot }095}\;\left(R^{2}=0_{\cdot }90\right) & \left(\text{Wet}\;\text{sack}\;\text{curing}\right)\\ \begin{array}{c} 1_{\cdot }040+0_{\cdot }080{\left(p\right)}^{0_{\cdot }904}+0_{\cdot }30{\left(t\right)}^{0_{\cdot }613}\;\left(R^{2}=0_{\cdot }84\right)\\ 1_{\cdot }978+0_{\cdot }225{\left(p\right)}^{0_{\cdot }610}+0_{\cdot }11{\left(t\right)}^{0_{\cdot }751}\;\left(R^{2}=0_{\cdot }98\right) \end{array} & \begin{array}{c} \left(\text{Curing}\;\text{compound}\right)\\ \left(\text{Steam}\;\text{curing}\right) \end{array} \end{array}\right.$$

Where p is the pressure (MPa) and t is the time of exerting pressure (hr).

**Fig. 14:**
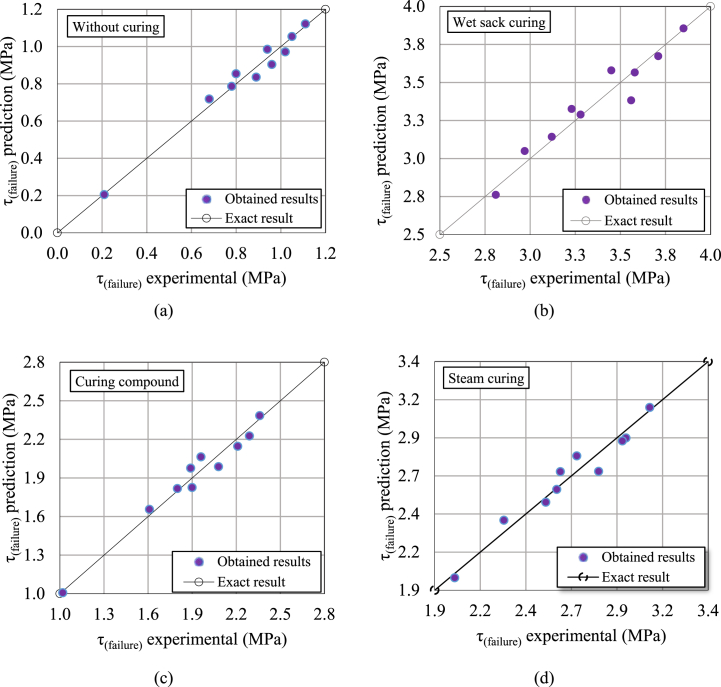
Comparison of predicted and experimental failure torsional shear stress between compressed HSRM and substrate HSC (a) without curing, (b) under wet sack curing, (c) using curing compound, (d) under steam curing, in twist-off test.

**Fig. 15:**
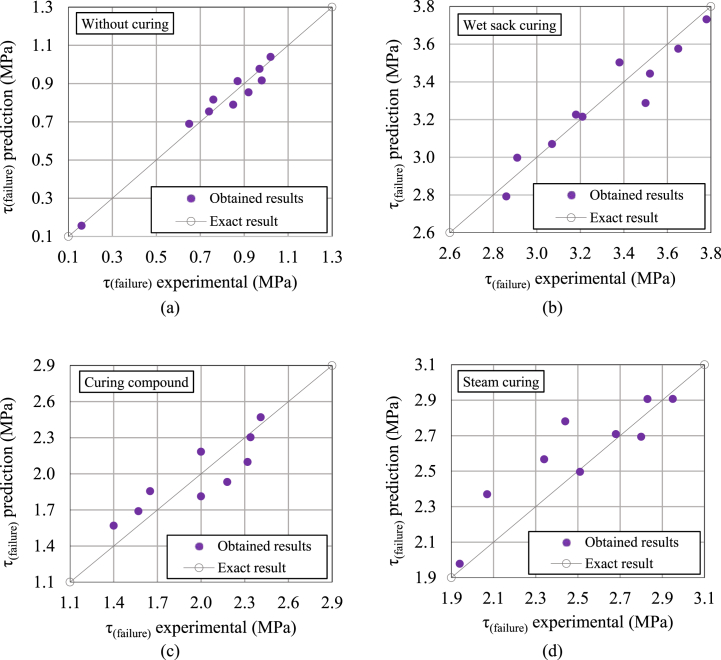
Comparison of predicted and experimental failure torsional shear stress between compressed HSRM and substrate HSC (a) without curing, (b) under wet sack curing, (c) using curing compound, (d) under steam curing, in friction transfer test.

According to the aforementioned equations, increasing both the pressure and the duration of pressure application led to an increase in the failure torsional shear stress between compressed HSRM and substrate HSC. For all proposed equations, the R^2^ coefficient was higher than 0.84, indicating a suitable prediction of the parameters. Fig. 14, Fig. 15 illustrate the comparison between the predicted and experimental results, demonstrating that the predicted values closely align with the diagonal line.

## 7 Conclusion

As highlighted in previous sections, practical application of HSC is limited due to its’ low workability and high durability and mechanical properties sensitivity to curing conditions. This study introduced a novel technique aimed at overcoming the workability barrier of HSC while simultaneously enhancing its bonding strength under various curing conditions. To achieve this, the combined effects of pressure and curing regimes on the modulus of elasticity and compressive strength of HSC and bonding strength between the substrate HSC and the 28-day HSRM were assessed. According to the results, the subsequent conclusions were drawn.

1. By increasing the pressure on HSC up to 5.23 MPa and exerting time was increased up to 8 h, the compressive strength and modulus of elasticity of HSC was enhanced on average 60 % and 61 %, respectively. Also, in this condition, the ultimate torsional shear strength between HSRM and substrate HSC was enhanced on average 174 %. The rate of failure torsional shear stress was high in pressure time of 2 h and then, was gradually decreased once the pressure time was continued for 8 h. Also, the modulus of elasticity and compressive strength of HSC increased considerably up to 4 h of pressure application, after which the rate of increase gradually diminished over the subsequent 4 h.
2. The bonding strength between HSRM and substrate HSC in the cases of using curing compounds and without curing was highly affected by time of pressure. While, in the cases of steam curing and wet sack curing, the bonding strength was highly affected by magnitude of pressure. Among the implemented curing methods, wet sack curing was the best curing method for enhancing the mechanical properties of HSC and HSRM specimens. Then, the steam curing and using curing compound were respectively resulted in lower failure torsional shear stress of compressed HSRM specimens.
3. According to the microstructural analysis, the microstructure of the uncompressed HSRM specimen without curing was more porous than that under wet sack curing, with larger voids on average found in the specimen without curing. Also, the microstructure of the compressed HSRM under wet sack curing was significantly denser than that of the uncompressed HSRM specimen.
4. The accuracy of predicted results indicate that the proposed equations are suitable for predicting the failure torsional shear stress of compressed HSRM specimens.

## 8 Limitations of the study

This study introduces a novel technique designed to overcome the low workability barrier of HSC while simultaneously improving its bonding strength under varying curing conditions. However, the magnitude and duration of pressure were limited. Thus, it is recommended to apply different pressures and durations under more curing methods such as water curing, microwave curing, and isothermal curing. Also, the effect of surface roughness of substrate concrete should be evaluated. Finally, it was recommended to evaluate the bonding performance of HSRM with different mixture designs.

## CRediT authorship contribution statement

**Parisa Bahri:** Writing – review & editing, Writing – original draft, Visualization, Validation, Resources, Methodology, Investigation, Formal analysis. **Mahmoud Naderi:** Supervision, Project administration, Conceptualization.

## Data and code availability statement

No data was used for the research described in the article.

## Declaration of competing interest

The authors declare that they have no known competing financial interests or personal relationships that could have appeared to influence the work reported in this paper.
